# A novel PBP3 substitution in *Haemophilus influenzae* confers reduced aminopenicillin susceptibility

**DOI:** 10.1186/s12866-018-1196-6

**Published:** 2018-05-31

**Authors:** John Thegerström, Erika Matuschek, Yu-Ching Su, Kristian Riesbeck, Fredrik Resman

**Affiliations:** 1 Clinical Microbiology, Department of Translational Medicine, Lund University, Jan Waldenströms gata 59, SE-205 02 Malmö, Sweden; 2 EUCAST Development Laboratory, Central Hospital Växjö, SE-351 85 Växjö, Sweden

**Keywords:** Ampicillin - β-lactam resistance - *ftsI* - *Haemophilus influenzae* - PBP3 - penicillin binding proteins - site directed mutagenesis

## Abstract

**Background:**

Identification and characterization of non-typeable *Haemophilus influenzae* (NTHi) with reduced susceptibility to β-lactam antibiotics due to mutations in penicillin binding protein 3 (PBP3) is a clinical challenge. We analyzed a blood isolate, NTHi93–57485, that was categorized as aminopenicillin resistant but lacked key amino acid substitutions in PBP3 that have previously been associated with reduced aminopenicillin susceptibility. The significance of an alternative amino acid substitution (Y528H) in this isolate was examined.

**Results:**

Site-directed mutagenesis of a β-lactam susceptible *H. influenzae* (NTHi3655) was performed to introduce the Y528H substitution into wild-type *ftsI* (encoding for PBP3). Disc diffusion screening and broth microdilution determination of MICs for β-lactam agents were done with the NTHi3655-PBP3^Y528H^ mutant and were compared with the NTHi3655 wild-type as well as the original clinical isolate NTHi93–57485. Introduction of the Y528H substitution in NTHi3655 resulted in positive screening for β-lactam resistance. MICs for aminopenicillins were increased in the mutant compared to the wild-type. However, the mutant remained susceptible to aminopenicillins according to EUCAST clinical breakpoints (assuming intravenous treatment) and the introduction of Y528H alone did not increase the resistance to the same level as NTHi93–57485. None of the isolates had frame shift insertions in the *acrR* gene regulating the AcrAB efflux pump.

**Conclusions:**

In parallel to the previously well-described PBP3-substitutions R517H and N526K, we demonstrate that Y528H confers reduced aminopenicillin susceptibility.

**Electronic supplementary material:**

The online version of this article (10.1186/s12866-018-1196-6) contains supplementary material, which is available to authorized users.

## Introduction

Antimicrobial resistance of the respiratory tract pathogen non-typeable *Haemophilus influenzae* (NTHi) to β-lactam antibiotics is conferred either by the production of transferrable β-lactamases or by amino acid substitutions in penicillin binding protein 3 (rPBP3), caused by point mutations of the *ftsI* gene [1]. It has also been shown that loss of repression of the AcrAB efflux pump in combination with rPBP3 may lead to a further increase in resistance [2].

NTHi strains with rPBP3 variants are classified into three main groups (Table 1), based on the substitution of two key amino acids occurring near the KTG-motif: R517H (clustered as group I) or N526K (group II) [3]. A third group with additional substitutions near the SSN-motif, S385T (group III or III-like) confers a higher-level of antimicrobial resistance, including resistance to third-generation cephalosporins [3–5]. The group II-rPBP3 variants can be further categorized into the subgroups IIa-d or A-G, depending on the pattern of mutations within *ftsI* that appear together with N526K [6, 7]. The evidence of correlation between these key substitutions and resistance phenotype is strong [3, 8], but the causal evidence of these substitutions as single determinants of resistance is less convincing. When Osaki and co-authors applied site-directed mutagenesis to introduce PBP3 substitutions into a β-lactam susceptible strain (*H. influenzae* Rd), neither the introduction of R517H nor N526K could alone generate mutants that were aminopenicillin resistant (ampicillin MIC = 0.25 mg/L for N526K) according to clinical breakpoints, although a reduced susceptibility compared to the wild-type (WT) isolate was seen [9].

**Table 1:** The principal groups of rPBP3 in *Haemophilus influenzae* with their associated amino acid substitutions and susceptibility to ampicillin. The clinical breakpoint for ampicillin is definied as *R* > 1 mg/L by EUCAST, which means that a subset of NTHi with rPBP3 genotype are still categorized as susceptible. Table modified after Skaare et al. [7]

Main rPBP3 group	Subgroup according to Skaare [7]	Subgroup according to Ubukata [3] and Dabernat [6]	Key amino acid substitutions	Associated substitutions in subgroups	MIC range of Ampicillin (mg/L)
Group I			R517H		0.5–2 [5]
Group II			N526K		0.5–8 [5]
	A	IIb	N526K	D350N M377I A502V V547I N569S	
	B	IId	N526K	I449V V547I N569S	
	C	IIb	N526K	D350N M377I G490E A502V V547I N569S	
	D	II-	N526K	D350N G490E A530S	
	E	IIc	N526K	A502T	
	F	IIa	N526K	–	
	G	II-	N526K	V547I A554T A561E N569S	
Group III			S385T + N526K		1–32 [4]
Group III-like			S385T + R517H		0.5–2 [5]

A screening algorithm to identify rPBP3 strains in routine diagnostics based on disc diffusion with 1 U benzylpenicillin is suggested by the European Committee on Antimicrobial Susceptibility Testing (EUCAST) [10]. This screening algorithm has demonstrated high sensitivity and specificity in detecting rPBP3 isolates, although confirmatory testing of actual MIC levels is recommended to determine if screening-positive isolates are actually resistant [8, 11]. Since current clinical breakpoints for aminopenicillins split the rPBP3 group, this implies that a subset of rPBP3 isolates are still considered susceptible [8] (Table 1). Moreover, the breakpoints assume intravenous dosage, and therefore there is debate on the optimal treatment of these strains in infections that do not require intravenous antibiotic therapy.

In the present study, we investigated a clinical NTHi isolate that was aminopenicillin resistant according to initial disc diffusion screening and MIC determination, but lacked resistance-defining substitutions in PBP3, and instead had an alternative substitution near the KTG-motif; Y528H.

## Methods

### Bacterial strains and culture conditions

**Fig. 1:**
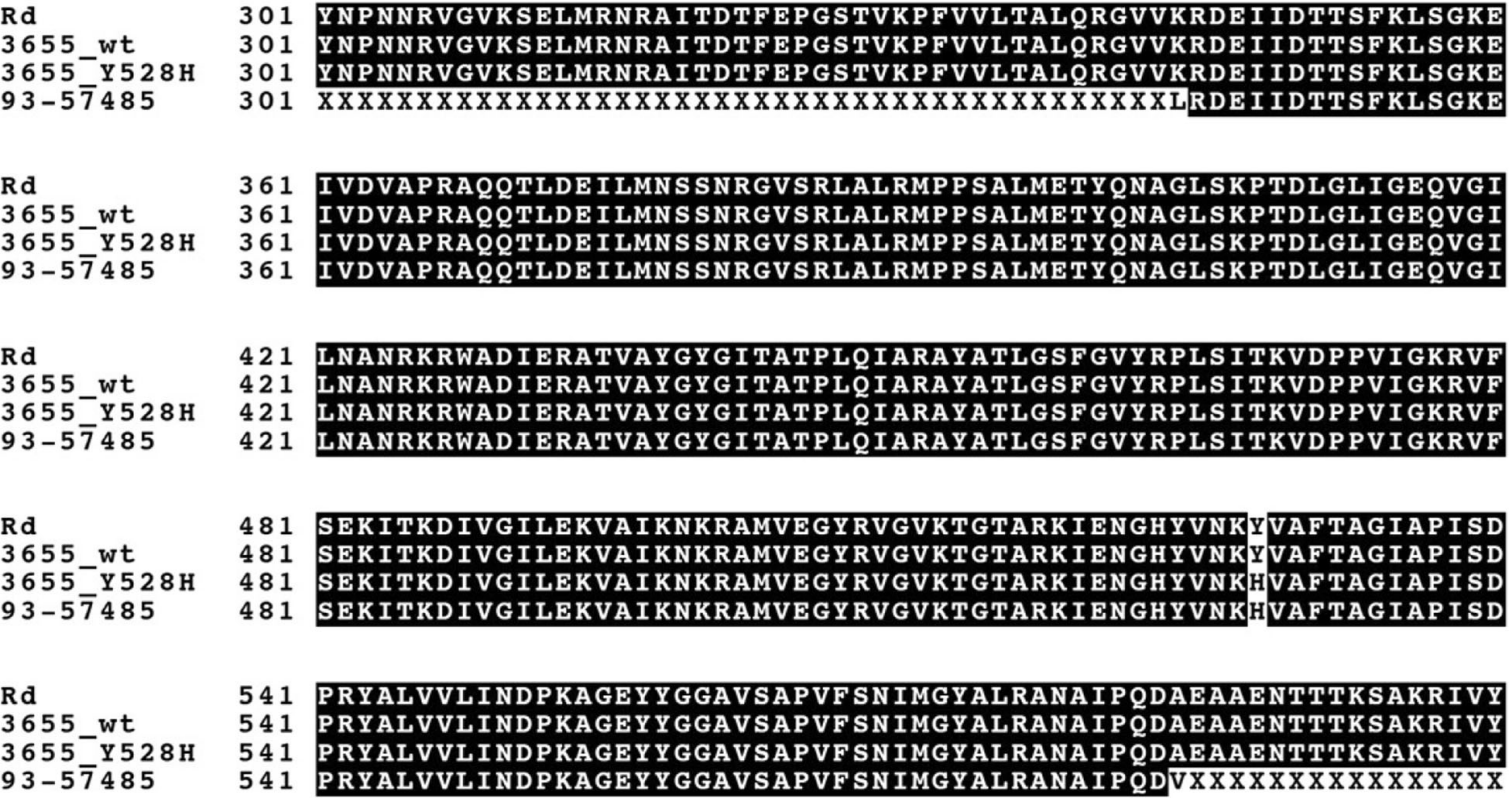
The amino acid sequence of the transpeptidase domain of PBP3 is shown for the wild-type PBP3 strain NTHi3655 as well as for the mutated strain NTHi3655-PBP3^Y528H^ and the clinical strain NTHi93–57,485 expressing the Y528H substitution. For comparison, the PBP3 sequence of *H.influenzae* Rd. is also shown (GenBank:U32793). None of the other resistance-associated substitutions listed in Table 1 is present in any of the isolates

A clinical blood isolate from Kronoberg County (Sweden) (NTHi93–57485) was collected as part of routine diagnostics at the laboratory of clinical microbiology in Växjö, Sweden. The isolate was screened as β-lactam resistant by disc diffusion, was β-lactamase negative by nitrocefin testing and had an MIC for amoxicillin of 2 mg/L according to the initial Etest (bioMérieux, Marcy l’Etoile, France), and thus aminopenicillin resistant according to EUCAST clinical breakpoints at the time (2011). The isolate did not, however, carry any of the previously described key mutations in *ftsI* and was therefore chosen for further testing. DNA sequencing of *fstI* revealed an alternative amino acid substitution located near the KTG-motif; Y528H (Fig. 1).

For site-directed mutagenesis, a well-characterized, β-lactam-susceptible isolate (NTHi3655) previously kindly donated by R. Munson, St Louis, Mo., was chosen [12]. This strain was chosen since its full genome sequence is known and it has a wtPBP3 sequence identical to that of *H.influenzae* Rd. (accession no. AAZF01000004.1). ATCC49766 (American Type Culture Collection, LGC standards, Teddington, UK) was used as quality control in the antimicrobial susceptibility testing. All strains were cultured on chocolate agar or in brain heart infusion (BHI) broth supplemented with 10 μg/ml each of nicotinamide adenine dinucleotide (NAD) and hemin overnight at 37°C and 5% CO_2_. All isolates were confirmed as *Haemophilus influenzae* by Matrix Assisted Laser Desorption Ionization Time of Flight (MALDI-TOF, scores > 2).

Only microorganisms and no human material were handled in this project.

### Site-directed mutagenesis (SDM)

Genomic DNA was purified using the GenElute™ Bacterial Genomic DNA kit (Sigma-Aldrich, St Louis, MO). The *ftsI* gene and its flanking regions were amplified from the WT NTHi3655 by using the Expand™ High Fidelity PCR System (Roche, Mannheim, Germany) and primers listed in Table 2.

**Table 2:** Primers used for PCR amplification, site directed mutagenesis and sequencing of the *ftsI* and *acrR* genes

Use of primers	Forward primer sequence	Reverse primer sequence
Ampification of full length *ftsI* gene	5′ – CCTGCGTGTTTGAAAGTTGAAAGAGATG – 3’	5′ – AACAAAGTAAGGGCGAGGATATTCCCAAAG – 3’
Introduction of Y528H substitution^a^ in *ftsI* in NTHi3655	5′ – GAAAATGGACATTATGTAAATAAGCATGTGGCATTTACTGCGGG – 3’	5′ – CCCGCAGTAAATGCCACATGCTTATTTACATAATGTCCATTTTC – 3’
Amplification and sequencing of the *acrR* gene	5′ – TTGTGGGTTTACGGCTTACC – 3’	5′ – CCGATGACACCGACAAAAAT – 3’
Sequencing of *ftsI* gene fw1 (using primer walking)	5′ – CCAATAAACTCTACAGTTAAATGCTCGC – 3’	
Sequencing of *ftsI* gene fw2 (using primer walking)	5′ – AGCGGACGATAAACACCGAAACTACCA – 3’	
Sequencing of *ftsI* gene fw3 (using primer walking)	5′ – ATACTTAAGGTAACATCTTGTGCATCATAT – 3’	

^a^ Induces nucleotide substitution 1582 T > C to change the codon from a tyrosine residue (TAT) to a histidine residue (CAT)

The resulting PCR product was cloned into the pCR-XL-TOPO^®^ vector by using TOPO^®^ XL PCR cloning kit (Invitrogen, Carlsbad, CA). The recombinant plasmid construct was thereafter transformed into *Escherichia coli* TOP10. The *fstI* gene was verified by DNA sequencing (Eurofins Genomics, Ebersberg, Germany). Site-directed mutagenesis was carried out using Pfu Turbo DNA polymerase (Agilent, Santa Clara, CA) and primers outlined in Table 2. The PCR products were digested using *Dpn*I (Thermo Scientific, Waltham, MA) for 1 h at 37 °C. The mutated *ftsI* gene with confirmed mutation of Y528H was amplified by PCR and transformed into the recipient strain NTHi3655 using the protocol by Poje and Redfield [13]. The generated mutant (named NTHi3655-PBP3^Y528H^) was selected on BHI agar containing NAD and hemin, and increasing concentrations of ampicillin (0, 0.125, 0.25, 0.5, 0.75, 1 and 2 mg/L, respectively). Finally, the *ftsI* gene sequence in the resulting mutant was verified by DNA sequencing.

### Antimicrobial susceptibility testing

Antimicrobial susceptibility testing was performed at the EUCAST development laboratory (Växjö, Sweden). Screening for β-lactam resistance with disc diffusion using 1 U of benzylpenicillin (PcG) on fastidious Mueller Hinton (MH-F) solid medium was performed with NTHi93–57,485, NTHi3655 and NTHi3655-PBP3^*Y528H*^ [10]. MICs to common β-lactam agents were also determined by broth microdilution (BMD) according to the ISO standard 20776–1 [14] using MH-F broth [15]. Absence of β-lactamase production was confirmed by a standard nitrocefin test [16].

### Growth curves and sequencing of the *acrR* gene

A few colonies of NTHi were resuspended in supplemented BHI broth and diluted to a starting OD_600_ of 0.05. The bacterial suspension was incubated at 37°C and 5% CO_2_ at 200 rpm and OD_600_ was measured at indicated time points. The *acrR* gene, which encodes a regulator of the AcrAB efflux pump was sequenced using primers stated in Table 2.

## Results

Site directed mutagenesis of the susceptible strain NTHi3655 yielded a mutant (NTHi3655-PBP3^Y528H^) with an identical transpeptidase PBP3 sequence to that of the clinical isolate NTHi93–57485 (Fig. 1). The introduction of the substitution Y528H into a wtPBP3 rendered the mutant NTHi3655-PBP3^Y528H^ positive in the disc diffusion β-lactam resistance screening algorithm (Table 3). The mutant also demonstrated a one- or two-fold increase in MICs for aminopenicillins as revealed by susceptibility testing with broth microdilution (Table 3). However, the clinical isolate NTHi93–57485 still had a higher MIC for ampicillin (1 mg/L) and cefuroxime (4 mg/L) compared with NTHi3655-PBP3^Y528H^. All strains were β-lactamase negative. None of the isolates had any frame shift insertions in the *acrR* gene.

**Table 3:** Results of screening for β-lactam resistance and susceptibility testing to various β-lactam antibiotics by BMD

Strain ID	Geno-type	Zone diameter PCG 1 U (mm)	Screening phenotype^a^	Susceptible to amino-penicillins^b^	MIC^c^ amoxicillin	MIC ampicillin	MIC amoxicillin clavulanic acid	MIC cefotaxime	MIC ceftriaxone	MIC cefuroxime	MIC imipenem	MIC mero-penem	β-lactamase
NTHi3655	Wild-type	16	Susceptible	Susceptible	0.5	≤0.25	≤0.25	≤0.015	≤0.015	1	0.5	0.06	Negative
NTHi3655-PBP3^Y528H^	Y528H	11	Resistant	Susceptible	1	0.5	1	≤0.015	≤0.015	1	1	0.06	Negative
NTHi93–57485	Y528H	6	Resistant	Susceptible	1	1	1	0.06	≤0.015	4	0.5	0.06	Negative

^a^ As interpreted by EUCAST clinical breakpoints for benzylpenicillin 1 U screening with a zone diameter < 12 mm categorized as resistant

^b^ Interpreted by EUCAST clinical breakpoints where ampicillin MIC > 1 mg/ L and Amoxicillin MIC > 2 mg/ L are categorized as resistant (assuming i.v. treatment)

^c^ MIC in mg/ L determined by BMD

According to BMD, all isolates had MICs that were below current clinical breakpoints proposed by EUCAST, contrary to the initial Etest results obtained with NTHi93–57485. It has previously been suggested that gradient tests have a tendency to overestimate MICs in *H. influenzae* with modified PBP3 [17].

## Discussion

The introduction of the amino acid substitution Y528H rendered a fully susceptible isolate to become positive in the benzylpenicillin screening test. It did not, however, restore zone or MIC levels of ampicillin to those of the clinical isolate NTHi93–57485. These results mimic the findings from site-direction mutagenesis experiments performed on substitutions N526K and R517H by Osaki et al. [9]. Our results further support the observation that mechanisms other than rPBP3, β-lactamase production or dysregulation of the AcrAB efflux pump affect susceptibility to β-lactams in *H. influenzae*. Interestingly, we also noted a reduced growth rate in NTHi93–57485 (Additional file 1) compared with NTHi3655 and NTHi3655-PBP3^Y528H^. Decreased fitness as shown by slower growth rates has been shown to correlate with antimicrobial resistance in other bacterial species [18].

When Osaki et al. introduced the key residue substitution of N526K into the PBP3 of *H. influenzae* Rd. strain, ampicillin MIC increased only 1-fold, in good agreement with our current findings in NTHi3655-PBP3^Y528H^ [9]. Therefore, despite the fact that these two main mutations near the KTG-motif managed to reduce aminopenicillin susceptibility, it seems that additional factors (PBP3-related or unrelated) are required for resistance surpassing clinical breakpoints. However, the prevalence of the substitution Y528H in clinical isolates seem to be distinctly lower compared with N526K. To the best of our knowledge, the Y528H substitution has only been sporadically described in studies where PBP3 has been sequenced, for instance, in two cefuroxime–resistant isolates where it appeared together with N526K and S357N [19]. A BLAST-search on publicly available PBP3 sequences on NCBI only identified one other isolate with this mutation (accession no. BAZ92405.1). The Y528H substitution is not included in the PBP3 substitutions previously investigated by site directed mutagenesis [9]. The reasons why this mutation seems less prevalent remain to be elucidated. Its introduction into PBP3 did not affect the growth rate of our experimental strain, but other manifestations of reduced bacterial fitness caused by this mutation still have to be conclusively ruled out. Also, it cannot be ruled out that the studied mutation is less efficient and thus less prone to selection by antibiotic treatment. It can be added to this discussion that the N526K substitution is rarely seen as a lone substitution in PBP3 in clinical isolates with reduced susceptibility to aminopenicillins.

Even though additional factors may be needed for resistance according to clinical breakpoints, several prior studies have demonstrated the importance of alterations in PBP3 for the development of β-lactam resistance in *Haemophilus influenzae* [3, 4, 9]. Group II strains, with a low-level aminopenicillin resistance, dominate in most studies [1, 7, 20]. 3D modelling has previously suggested that the N526K substitution lines the active site pocket of PBP3, near the catalytic motif of KTG-514 [3]. Given its proximity to this motif, it is likely that the Y528H substitution also interferes with the active site pocket.

The proportional increase in recent years in rPBP3 strains together with increasing rates of resistance to sulfamethoxazole limits the number of treatment options for common respiratory tract infections, especially in children [20]. Also, current breakpoints for aminopenicillins assume intravenous dosage, whereas in everyday treatment of less severe infections, oral amoxicillin is often used. Pharmacokinetic simulations suggest that even a high oral amoxicillin dose (750 mg tid) does not always result in 40% *f*T > MIC (including 2 standard deviations) for an isolate with an MIC of 1 mg/L [21]. Due to this, there is debate whether low-rPBP3 strains that are considered as susceptible according to clinical breakpoints can be safely treated with oral amoxicillin in clinical practice, where a dose of 500 mg tid is commonly used. Until clinical studies have been performed to address this issue, there is a case for inclusive screening regimes.

## Conclusions

In conclusion, we have identified a novel PBP3-mutation, Y528H, that affects aminopenicillin susceptibility in *H. influenzae*, and this mutation should be added to rPBP3-defining substitutions. It is clear that mechanisms other than β-lactamase production, point mutations in PBP3 or dysregulation of the AcrAB efflux pump can contribute to reduced β-lactam susceptibility.

## Additional file

Additional file 1: Growth curves demonstrate that the clinical strain NTHi93–57,485 has a slower growth rate than the rPBP3 WT strain NTHi3655 and the corresponding mutant NTHi3655-PBP3^Y528H^. Mean values from 3 separate experiments are shown. Error bars represent standard error of the mean (SEM). Circles represent NTHi3655, squares represent NTHi3655-PBP3^Y528H^ and triangles represent NTHi93–57,485. (DOCX 72 kb)

## Abbreviations

BHI: brain heart infusion (broth and solid medium)
BMD: Broth Microdilution
EUCAST: European Committee on Antimicrobial Susceptibility Testing
*f*T > MIC: The amount of time in which free or non-protein-bound antimicrobial concentration exceeds the minimum inhibitory concentration (MIC) of the organism
MALDI-TOF: Matrix Assisted Laser Desorption Ionization - Time of Flight
MH-F: fastidious Mueller Hinton (broth and solid medium)
MIC: Minimal inhibitory concentration
NAD: nicotinamide adenine dinucleotide
NTHi: non-typeable *Haemophilus influenzae*
PBP3: Penicillin Binding Protein 3
PcG: Benzylpenicillin
rPBP3: PBP3 mediated resistance present (‘Resistant’ PBP3)
wtPBP3: wild type PBP3
